# Biochemical Monitoring of Antipsychotic Treatment in Children and Adolescents: A Retrospective Review within Maltese Child and Adolescent Mental Health Services

**DOI:** 10.1192/j.eurpsy.2026.10575

**Published:** 2026-07-31

**Authors:** N. Galea, D. V. Fondacaro

**Affiliations:** 1 Mater Dei Hospital, Msida; 2 Mount Carmel Hospital, Attard; 3 Department of Psychiatry, University of Malta, Msida, Malta

## Abstract

**Introduction:**

The prescription of antipsychotics in children and adolescents has increased significantly over the past decade, primarily for managing challenging behaviour and irritability in neurodevelopmental conditions such as ADHD and ASD (Radojčić et al. Lancet Psychiatry 2023; 10 119–128). Risperidone and aripiprazole are commonly used, but these drugs carry the risk of serious side-effects, particularly metabolic, cardiovascular, and hormonal. The NICE Quality Standard (QS102) recommends regular biochemical monitoring, but compliance with these guidelines in local practice remains unclear.

**Objectives:**

To evaluate adherence to the NICE-recommended biochemical monitoring protocols for children and adolescents prescribed antipsychotics in Maltese Child and Adolescent Mental Health Services (CAMHS).

**Methods:**

A retrospective case-note review of 350 patients attending CAMHS was conducted. One hundred patients prescribed antipsychotics for ≥9 consecutive months were included. Data was extracted from the iCM system to determine whether HbA1c or fasting glucose, lipid profile, and serum prolactin were assessed at baseline, 12 weeks, and 9 months, in line with the NICE Quality Standard.

**Results:**

No patient underwent all recommended monitoring at the specified intervals. Baseline monitoring occurred in 0%, 12-week monitoring in 1%, and 9-month monitoring in 2% of patients. At least two-thirds of the recommended tests were completed at any time point in 28% of cases. Risperidone (56%) and aripiprazole (42%) were the most commonly prescribed agents, with quetiapine (2%) being prescribed for the remainder. The majority of patients were diagnosed with ADHD (47%) or ASD (37%). Regarding biological sex, females accounted for 16% of the sample, while males accounted for 84%.

**Table 1.** Diagnostic Characteristics of Patients Prescribed Antipsychotics (n = 100) Table 1. Long description.

Variable	n (%)
**Primary Diagnosis**	
ADHD	47 (47%)
ASD	37 (37%)
ADHD + ASD	10 (10%)
Other*	6 (6%)

*Other: Global developmental delay, oppositional defiant disorder, learning disability, personality disorder traits, psychosis, anxiety, bipolar disorder.

**Image 1:**
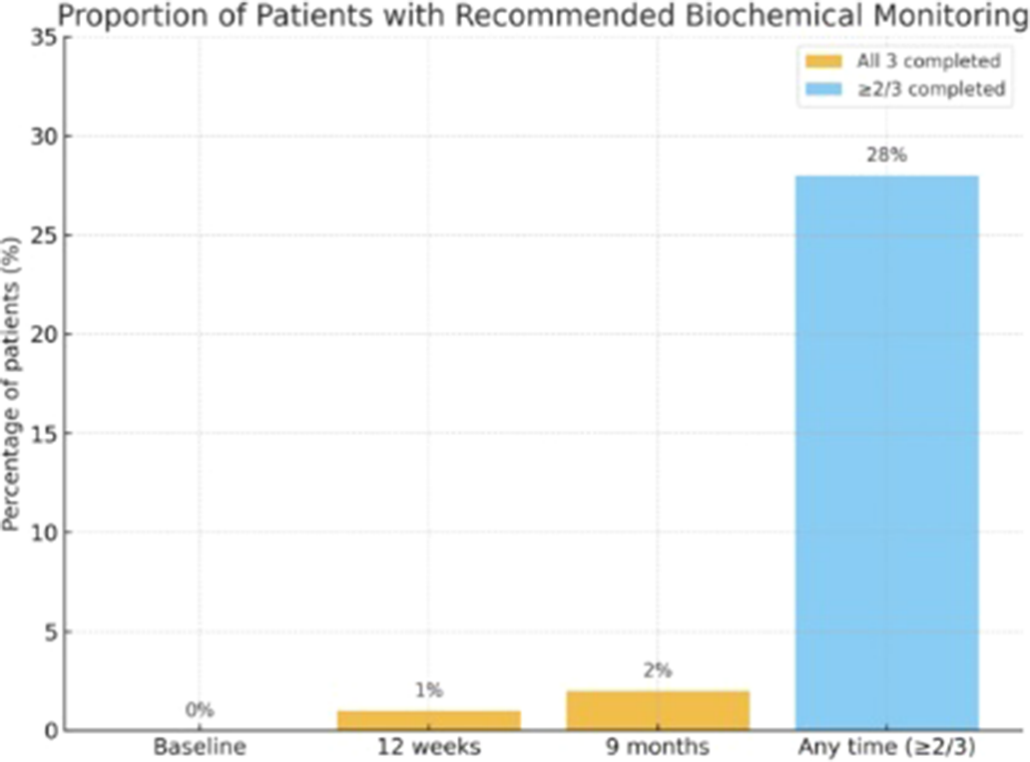

**Conclusions:**

Biochemical monitoring of antipsychotic use in Maltese CAMHS does not align with international standards. Interventional strategies including the creation of evidence-based protocols, clinician teaching, and integration of monitoring reminders into electronic systems should be considered. Improving surveillance of side-effects, not just biochemical, will strengthen patient safety and the quality of care; however, any overarching reasons why biochemical tests were not monitored should also be considered, including the risk/benefit profile and the potential psychological ramifications of blood-letting in young people. Opportunities exist for further research, including a re-audit in one year, and surveilling the monitoring of all side-effects.

**Disclosure of Interest:**

None Declared

## Long descriptions

### Table 1. Long description

The table consists of two columns: Variable and n (%).

Under the Primary Diagnosis header, the data is distributed as follows:

- A D H D: 47 (47%)

- A S D: 37 (37%)

- A D H D + A S D: 10 (10%)

- Other: 6 (6%)

A footnote specifies that Other includes global developmental delay, oppositional defiant disorder, learning disability, personality disorder traits, psychosis, anxiety, and bipolar disorder.

Navigate back to Table 1..

